# Causal associations between plasma metabolites and head and neck cancer: a bidirectional Mendelian randomization study

**DOI:** 10.1016/j.bjorl.2026.101812

**Published:** 2026-04-28

**Authors:** Zhaoyu Pan, Xueying Wang, Ziyuan Xu, Mingshui Lu, Xin Zhang, Lei Xiao

**Affiliations:** a Department of Otolaryngology Head and Neck Surgery, The Affiliated Children’s Hospital of Xiangya School of Medicine, Central South University (Hunan Children’s Hospital), Changsha, China; b Central South University, Department of Otolaryngology Head and Neck Surgery, Xiangya Hospital, Changsha, China; c Otolaryngology Major Disease Research Key Laboratory of Hunan Province, Changsha, China

**Keywords:** Head and neck cancer, Genome-Wide Association Study (GWAS), Causal inference, Plasma metabolites, Mendelian randomization

## Abstract

**Objective:**

Although observational studies have reported associations between plasma metabolites and Head and Neck Cancer (HNC), their causal relationships remain unclear. This study aimed to systematically evaluate the causal associations between circulating plasma metabolites and HNC risk using a bidirectional Mendelian Randomization (MR) approach.

**Methods:**

We conducted a bidirectional two-sample MR analysis leveraging Genome-Wide Association Study (GWAS) summary statistics for 1400 plasma metabolites and HNC from the FinnGen consortium. Causal estimates were primarily derived using the Inverse Variance-Weighted (IVW) method, complemented by MR-Egger, weighted median, and weighted mode approaches. Sensitivity analyses, including heterogeneity tests, pleiotropy assessment, and leave-one-out analyses, were performed to ensure robustness. To validate the findings, significant associations were replicated using independent HNC GWAS data from the IEU OpenGWAS database.

**Results:**

IVW analysis identified three plasma metabolites causally associated with increased HNC risk: 1,2-dipalmitoyl-gpc (16:0/16:0) (OR = 1.20, 95% CI 1.06–1.37, p = 0.004), P-cresol glucuronide (OR = 1.22, 95% CI 1.03–1.45, p = 0.023), and Nisinate (24:6n3) (OR = 1.15, 95% CI 1.01–1.32, p = 0.038). These associations were directionally consistent and supported by sensitivity and replication analyses. No evidence suggested reverse causality from HNC to these metabolites.

**Conclusions:**

This study provides systematic genetic evidence supporting causal roles of specific plasma metabolites in HNC development. These findings enhance the understanding of metabolic mechanisms underlying HNC and may inform future biomarker discovery and preventive or therapeutic strategies.

**Level of evidence:**

Level 3.

## Highlights

• This study establishes a link between plasma metabolites and the risk of head and neck cancer.
• 1,2-dipalmitoyl-gpc (16:0/16:0), P-cresol glucuronide, and Nisinate (24:6n3) can improve the risk of head and neck cancer.
• Targeting plasma metabolites can offer strategies for head and neck cancer’s treatment.

## Introduction

Head and neck cancer is the sixth most common cancer in the world, and the incidence rate is increasing year by year, with up to 890,000 new cases and 450,000 deaths annually worldwide.^1^ It is primarily squamous cell carcinoma and originates from the epithelial cells of the mucous membranes of the oral cavity, pharynx, larynx, etc.^2^ The treatment approach is often based on a combination of surgery, chemotherapy, and radiation, after which many patients are confronted with swallowing and speech impairments.^3^ Although targeted immunotherapy has improved some survival rates in recent years, only a minority of patients have a durable response, and recurrence and metastasis remain pressing problems.^4^

Metabolic reprogramming is one of the hallmarks of cancer and confers the potential for cancer cells to grow and proliferate in a nutrient-deficient tumor microenvironment.^5^ Tumor cells autonomously alter their fluxes through various metabolic pathways to meet increased bioenergetic and biosynthetic demands and to mitigate the oxidative stress required for tumor cell proliferation and survival.^6^ Because metabolic enzymes are attractive therapeutic targets for cancer therapy, a more comprehensive identification of critical metabolic targets and more precise tracking of tumor metabolic reprogramming can lead to more personalized metabolic therapy.^7^ Observational studies have revealed that glycolysis, tricarboxylic acid cycle, and glutamine metabolism are upregulated in head and neck cancer tissues.^8^^,^^9^ Moreover, previous researches have demonstrated the tumor-suppressing function of coenzyme Q0 and the tumor-promoting function of kynurenine in head and neck cancer.^10^^,^^11^ However, limited plasma metabolites have been studied to date, so the exact role and contribution of metabolites in this disease still need to be further explored. Furthermore, the causal association between plasma metabolites and head and neck cancer remains unclear.

Mendelian Randomization (MR) is a type of study that uses genetic variation associated with exposures to assess the possible causal relationship with outcomes.^12^ Because alleles follow the principle of random assignment, MR is similar to the randomized controlled trial and can reduce the potential bias of confounding and reverse causation in observational epidemiological studies.^13^

In this study, we take the lead in using a bidirectional MR analysis to investigate the potential causal association between a network of 1400 plasma metabolites and the risk of head and neck cancer, which could provide new insights into the medical management of such disease.

## Methods

### Study design

**Fig. 1:**
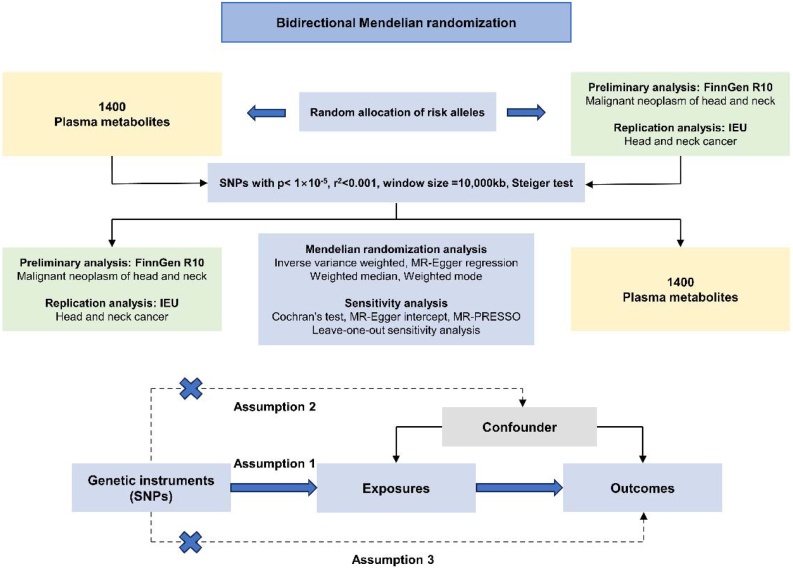
Study design overviews and assumptions of Mendelian randomization analysis.

To delve into the causal effect of 1400 plasma metabolites on head and neck cancer, a bidirectional two-sample MR analysis was performed according to the model shown in Fig. 1. The analysis process follows three key assumptions: (1) The Instrumental Variables (IVs) must be robustly correlated with the exposure; (2) The IVs are not related to confounders; (3) The IVs affect the outcome only through the exposure.^14^ Ethical approval can be available from previously published studies, and this MR study abode by the reporting specification of Strengthening the Reporting of Observational Studies in Epidemiology using Mendelian Randomization (STROBE-MR) in Table S1.

### Data sources

Plasma metabolite data was derived from the most comprehensive Genome-Wide Association Study (GWAS), containing 1,091 metabolites and 309 metabolite ratios from 8,299 European individuals.^15^ These metabolites cover eight major pathways including carbohydrate, lipid, nucleotide, amino acid, cofactor and vitamins, peptide, energy, and xenobiotics. The genetic association data for head and neck cancer originated from the FinnGen consortium (r10.finngen.fi), comprising 2,281 cases and 314,193 controls of Finnish ancestry, which was used for preliminary analysis. To our knowledge, these datasets do not overlap, and the detailed information can be found in Tables S2‒S3.

### The selection of IVs

To get enough Single Nucleotide Polymorphisms (SNPs), we eased the genome-wide significance threshold to p < 1 × 10^−5^ to select metabolite-associated SNPs. Independent SNPs were clumped by removing Linkage Disequilibrium (LD, *r*^2^ < 0.001 within a 10,000 kb window). For head and neck cancer, given the few significant IVs, we also adjusted the significance threshold to p < 1 × 10^−5^. Weak IVs were eliminated by calculating F-statistics < 10.^16^ In the process of harmonizing exposure and outcome data, palindromic SNPs were removed. Steiger test was used to inspect the reverse causality.

### Preliminary analysis

A bidirectional two-sample MR analysis was employed to evaluate the causal correlation between 1400 plasma metabolites and head and neck cancer. Several statistical methods were applied to this study, including Inverse Variance Weighted (IVW), MR-Egger regression, weighted median, and weighted mode.^17^, ^18^, ^19^ The application of IVW presupposes that all SNPs are valid IVs and are entirely independent of each other. The weighted median yields robust estimates when at least 50% of the information comes from valid IVs.

Different approaches have been chosen for sensitivity analysis, such as Cochran's *Q* test, MR-Egger intercept test,^20^ MR pleiotropy residual sum and outlier (MR-PRESSO), and leave-one-out sensitivity analysis.^21^ Cochran's *Q* test was adopted to assess heterogeneity, and a fixed-effect model was selected in the absence of significant heterogeneity. The horizontal pleiotropy was indicated in the MR-Egger intercept test when p-values for intercept < 0.05. MR-PRESSO can be used to obtain estimates that are closer to the actual value by excluding outliers. Leave-one-out sensitivity analysis progressively eliminated each SNP and observed whether the results changed significantly after the removal of each SNP. The above analyses were implemented using the TwoSampleMR (version 0.5.11) and MR-PRESSO (version 1.0) packages in the R platform (version 4.3.1).

### Replication analysis

To comprehensively validate the robustness of the candidate metabolites, we replicated the above analysis in the IEU Open dataset. The GWAS summary data for head and neck cancer obtained from the IEU Open dataset includes 1,106 cases and 372,016 controls of European ancestry with a sequencing depth of 9,655,080 SNPs (https://gwas.mrcieu.ac.uk/datasets/ieu-b-4912/).

## Results

All IVs used in the analysis had F-statistics greater than 10, demonstrating no weak instrument bias. Details on the selected IVs are displayed in Tables S4‒S7.

### Causal effects of metabolites on head and neck cancer

**Fig. 2:**
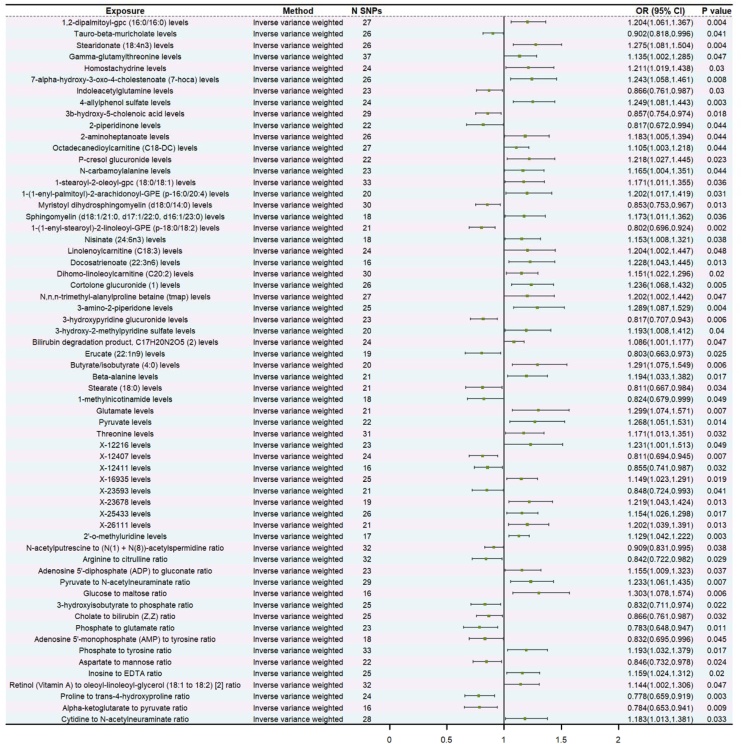
Forward Mendelian randomization analysis for causal effects of metabolites on head and neck cancer.

**Fig. 3:**
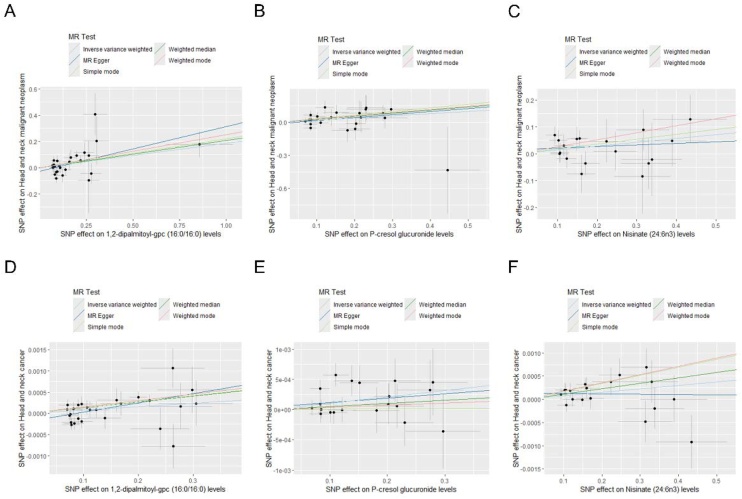
The scatter plots of preliminary analysis (A‒C) and replication analysis (D‒F).

In preliminary analysis, a total of 62 metabolites were identified that were associated with head and neck cancer from the FinnGen consortium, of which 40 metabolites were associated with an increased risk of head and neck cancer, and the remaining 22 metabolites were associated with a reduced risk (Fig. 2, Table S8). To further improve the persuasiveness of this analysis, we performed a replication analysis using the IEU dataset of head and neck cancer and identified 69 metabolites associated with it, of which 35 metabolites were risk factors for head and neck cancer and the remaining 34 were protective factors (Table S9). A subsequent joint analysis of FinnGen and IEU datasets showed that only three metabolites passed the replication test (Fig. S1). Based on IVW analysis, 1,2-dipalmitoyl-gpc (16:0/16:0) levels (OR = 1.20, 95% CI 1.06–1.37, p = 0.004), P-cresol glucuronide levels (OR = 1.22, 95% CI 1.03–1.45, p = 0.023), and Nisinate (24:6n3) levels (OR = 1.15, 95% CI 1.01–1.32, p = 0.038) were associated with an increased risk of head and neck cancer. The MR-Egger regression, weighted median, and weighted mode yielded consistent causal estimates on the trend (Fig. 3).

**Fig. 4:**
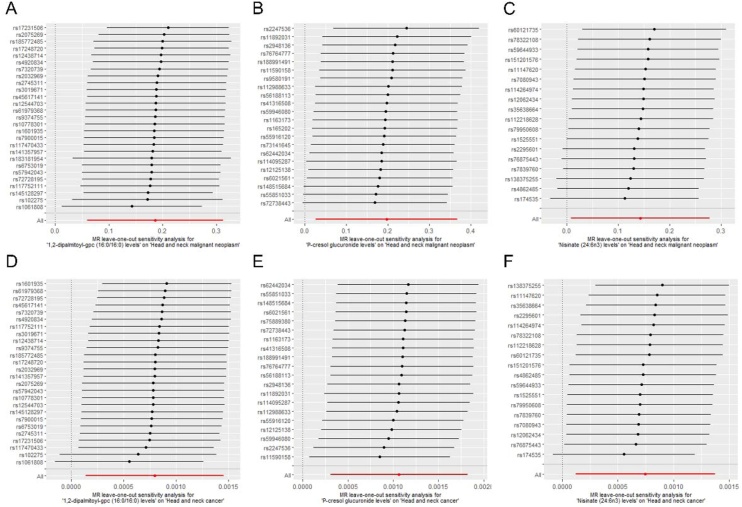
The leave-one-out analysis of preliminary analysis (A‒C) and replication analysis (D‒F).

In sensitivity analysis, Cochran's Q test verified the absence of heterogeneity, and MR-PRESSO did not detect outliers. MR-Egger intercept test of 1,2-dipalmitoyl-gpc (16:0/16:0) levels (intercept = −0.026, p = 0.104), P-cresol glucuronide levels (intercept = −0.010, p = 0.669), and Nisinate (24:6n3) levels (intercept = 0.015, p = 0.565) were suggestive of no horizontal pleiotropy (Table 1). Leave-one-out analysis confirmed that no individual SNP drove the bias (Fig. 4). The forest plots and funnel plots are displayed in Figure S2‒S3.

**Table 1:** Results of heterogeneity and pleiotropy assessment.

Outcome	Exposure	Cochran's *Q* test			MR-Egger intercept			MR-PRESSO	
		Method	*Q*	p-value	Intercept	Se	p-value	Global Test	p-value
Malignant neoplasm of head and neck	1,2-dipalmitoyl-gpc (16:0/16:0) levels	MR Egger	32.058	0.156	−0.02618	0.01550	0.104	11.024	0.743
		Inverse variance weighted	35.715	0.097					
Malignant neoplasm of head and neck	P-cresol glucuronide levels	MR Egger	19.296	0.503	−0.01019	0.02346	0.669	21.252	0.582
		Inverse variance weighted	19.485	0.554					
Malignant neoplasm of head and neck	Nisinate (24:6n3) levels	MR Egger	13.531	0.634	0.01510	0.02569	0.565	15.570	0.692
		Inverse variance weighted	13.876	0.676					
Head and neck cancer	1,2-dipalmitoyl-gpc (16:0/16:0) levels	MR Egger	30.511	0.168	−0.00016	0.00009	0.104	11.024	0.743
		Inverse variance weighted	36.233	0.068					
Head and neck cancer	P-cresol glucuronide levels	MR Egger	18.355	0.432	0.00004	0.00012	0.708	20.370	0.510
		Inverse variance weighted	18.503	0.489					
Head and neck cancer	Nisinate (24:6n3) levels	MR Egger	17.980	0.325	0.00014	0.00012	0.273	22.049	0.312
		Inverse variance weighted	19.428	0.305					
1,2-dipalmitoyl-gpc (16:0/16:0) levels	Malignant neoplasm of head and neck	MR Egger	0.865	0.990	0.01199	0.03796	0.763	1.233	0.996
		Inverse variance weighted	0.965	0.995					
P-cresol glucuronide levels	Malignant neoplasm of head and neck	MR Egger	1.018	0.998	0.00964	0.02046	0.650	1.488	1.000
		Inverse variance weighted	1.240	0.999					
Nisinate (24:6n3) levels	Malignant neoplasm of head and neck	MR Egger	0.352	0.986	−0.00004	0.02377	0.999	0.499	0.995
		Inverse variance weighted	0.352	0.997					
1,2-dipalmitoyl-gpc (16:0/16:0) levels	Head and neck cancer	MR Egger	1.447	1.000	−0.00045	0.01587	0.978	1.686	1.000
		Inverse variance weighted	1.448	1.000					
P-cresol glucuronide levels	Head and neck cancer	MR Egger	1.859	0.997	−0.00362	0.01759	0.841	2.258	1.000
		Inverse variance weighted	1.902	0.999					
Nisinate (24:6n3) levels	Head and neck cancer	MR Egger	0.094	0.999	0.01180	0.03899	0.777	0.269	0.998
		Inverse variance weighted	0.185	0.999

### Causal effects of head and neck cancer on metabolites

**Fig. 5:**
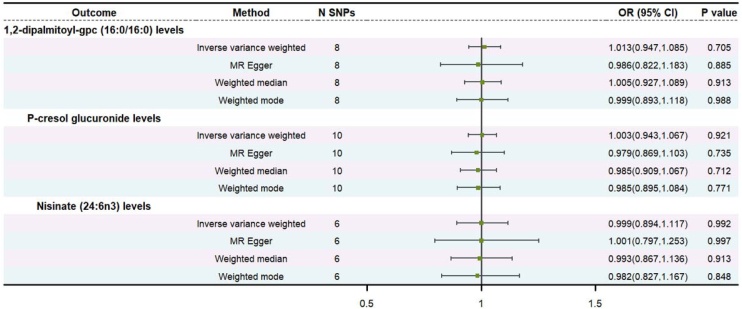
Reverse Mendelian randomization analysis for causal effects of head and neck cancer on metabolites.

To evaluate reverse causal effects, we conducted reverse MR analyses using head and neck cancer from the FinnGen or IEU dataset as the exposure and 1400 plasma metabolites as outcomes. The results showed no causal relationship between head and neck cancer and 1,2-dipalmitoyl-gpc (16:0/16:0) levels (OR = 1.01, 95% CI 0.95–1.09, p = 0.705), P-cresol glucuronide levels (OR = 1.00, 95% CI 0.94–1.07, p = 0.921), and Nisinate (24:6n3) levels (OR = 1.00, 95% CI 0.89–1.12, p = 0.992). Multiple methods of MR analysis drew accordant conclusion (Fig. 5, Tables S10‒S11). MR-Egger intercept test revealed no horizontal pleiotropy in 1,2-dipalmitoyl-gpc (16:0/16:0) levels (intercept = 0.012, p = 0.763), P-cresol glucuronide levels (intercept = 0.010, p = 0.650), and Nisinate (24:6n3) levels (intercept = −4.22E-05, p = 0.999). Cochran's *Q* test and MR-PRESSO verified the absence of heterogeneity and pleiotropy in the reverse analysis (Table 1).

## Discussion

In the current analysis, a bidirectional MR study was employed to integrate two large-scale datasets of head and neck cancer, exploring the causal role of 1400 plasma metabolites. We demonstrated that genetically predicted high levels of 1,2-dipalmitoyl-gpc (16:0/16:0), P-cresol glucuronide, and Nisinate (24:6n3) were associated with an elevated risk of head and neck cancer. The sensitivity analysis provided strong evidence for the robustness of the results. However, reverse MR analysis showed no significant causal association between head and neck cancer and 1400 plasma metabolites.

Recent years have witnessed substantial progress in the diagnosis and management of head and neck cancer, driven by advances in imaging technologies, molecular pathology, and precision medicine.^22^ High-resolution imaging techniques, including multiparametric magnetic resonance imaging and positron emission tomography-computed tomography, have significantly improved tumor localization, staging accuracy, and treatment planning.^23^ In parallel, molecular and genetic profiling is increasingly integrated into clinical practice, enabling refined risk stratification and personalized therapeutic decision-making. Contemporary diagnostic frameworks emphasize the combination of radiological assessment with histopathological and molecular markers to improve early detection and optimize treatment outcomes in head and neck cancer.^24^ Moreover, multidisciplinary management strategies ‒ incorporating surgery, radiotherapy, systemic therapy, and emerging targeted approaches ‒ have led to measurable improvements in tumor control and patient survival.^25^ These developments underscore the importance of identifying novel biomarkers that can complement existing diagnostic tools. In this context, elucidating the causal roles of plasma metabolites in head and neck cancer may contribute to the development of noninvasive biomarkers and support more precise diagnostic and therapeutic strategies.

Metabolic reprogramming, as one of the essential hallmarks of cancers, provides an important material and energy basis for the growth, proliferation, and metastasis of cancer cells.^6^ Metabolic reprogramming not only acts on cancer cells, but also regulates their growth and proliferation process by affecting the tumor microenvironment.^26^ Previous observational studies have shown that glucose metabolism, fatty acid metabolism, and amino acid metabolism play important roles in the proliferation and drug resistance of head and neck cancer. Still, the causal associations have not been elucidated.^27^^,^^28^ In this study, three metabolites were screened as risk factors for head and neck cancer by preliminary and replication MR analyses, providing new evidence for metabolic network awareness.

1,2-dipalmitoyl-gpc (16:0/16:0) is a phosphatidylcholine belonging to a type of glycerophospholipid. Phosphatidylcholine is both a major component of cell membranes and a source of energy.^29^ As a product of phospholipid metabolism, it is essential for membrane structural integrity as well as for lipid-dependent signaling pathways, and it is an essential component necessary for cancer cell growth.^30^ Phospholipid metabolites have important roles in tumor cell membrane fluidity, proliferation, invasion, and metastasis, so phospholipids have the potential to be used as diagnostic biomarkers or anticancer therapeutic targets.^31^^,^^32^ Researchers have identified significant differences in phosphatidylcholine in cancer and stromal regions of oral squamous cell carcinoma.^33^ However, 1,2-dipalmitoyl-gpc (16:0/16:0) has only been studied in lipid metabolism in oligodendrocytes but not in head and neck cancer.^34^ Hence our results provide new insights into its causal role in head and neck cancer.

P-cresol glucuronide is a glucuronide derivative, a type of p-cresol normally excreted in the urine. Current research focuses on its role as a uremic toxicity observed to cause an increased risk of cardiovascular disease in chronic kidney disease.^35^ In tumor-related studies, P-cresol glucuronide has been validated as a potential diagnostic biomarker for bladder cancer and a valuable staging biomarker for non-muscle-invasive bladder cancer.^36^ Another study elucidated that the metabolism of P-cresol glucuronide is increased in renal cell carcinoma and may serve as a potential diagnostic marker for renal cell carcinoma.^37^ Currently, there are no studies on the association of P-cresol glucuronide and head and neck cancer, so our findings provide clues for future exploration.

Nisinate (24:6n3), known as Nisinic acid or Tetracosahexaenoic acid, belongs to a class of very long chain polyunsaturated fatty acids. It is expressed at elevated levels in mammalian reproductive organs and retina and is involved in docosahexaenoic acid biosynthesis. The previous study has selected Nisinate as a candidate biomarker for cervical cancer by comprehensive analysis of metabolomics and transcriptomics.^38^ The recent research exploring key metabolite biomarkers for colorectal cancer by systematic MR analysis of plasma metabolites has shown that Nisinate is positively associated with the risk of colorectal cancer.^39^ The role of Nisinate in various cancers is similar to our results, and our study provides new evidence for future research.

While earlier epidemiological studies suggested that lipid-related metabolites, phenolic compounds, and polyunsaturated fatty acids may be involved in carcinogenesis, their conclusions were often limited by confounding factors and reverse causation.^40^ Our MR-based approach provides genetic evidence supporting a potential causal role of selected metabolites, thereby strengthening and extending prior observations. We further discussed the biological plausibility of our key findings. For instance, elevated levels of 1,2-dipalmitoyl-gpc (16:0/16:0) have been implicated in membrane remodeling and inflammatory signaling, processes known to facilitate tumor initiation and progression.^29^ Similarly, P-cresol glucuronide, a gut microbiota-derived metabolite, has been linked to systemic inflammation and epithelial dysfunction, which aligns with emerging evidence on the role of microbiome-related metabolites in cancer development.^41^ Additionally, Nisinate (24:6n3), a long-chain polyunsaturated fatty acid, has been less extensively studied in head and neck cancer, but recent studies indicate that specific lipid subclasses and metabolic contexts may exert divergent biological effects.^42^ Moreover, we contrasted our results with existing MR studies in other cancer types, emphasizing similarities in methodological approaches and differences in identified metabolites. This comparison underscores the uniqueness of the metabolic landscape in head and neck cancer and highlights the added value of our comprehensive metabolome-wide MR analysis.

There are several advantages to this bidirectional MR analysis. First, it is the most comprehensive MR study to date on plasma metabolites and head and neck cancer, involving a large sample size of 1400 plasma metabolites. Second, rigorous analysis was performed using multiple MR methods, such as the Steiger test, sensitivity analysis, and reverse MR analysis. The results of various MR methods were directionally consistent, and the sensitivity analysis showed that it was not affected by heterogeneity and horizontal pleiotropy, which increased the robustness of the results. Third, we chose the FinnGen dataset for the preliminary analysis and used the IEU dataset for the replication analysis to improve the reliability of the results. However, this study also has some limitations. Due to the limited number of SNPs for the exposure of interest, we slightly relaxed the p-value threshold, as previous studies have done. In addition, the GWAS datasets we chose were all from European ancestry, and whether our findings can be generalized to other ethnic groups still needs to be investigated in the next step.

## Conclusion

In summary, this bidirectional MR study reveals the causal role of three plasma metabolites in head and neck cancer. In addition, our study refines the knowledge of metabolite networks in head and neck cancer. It helps to find key metabolic markers, which can provide valuable insights for screening, diagnosis, and treatment of head and neck cancer from a metabolic perspective.

## ORCID ID

Xueying Wang: 0009-0006-4248-5175

Ziyuan Xu: 0009-0008-1420-0229

Mingshui Lu: 0009-0002-0943-8335

Xin Zhang: 0009-0001-4486-6661

## Funding

This study was supported by the project of Postgraduate Independent Exploration and Innovation of Central South University (nº 2023ZZTS0832).

## Data availability statement

The authors declare that all data are available in repository.

## Declaration of competing interest

The authors declare no conflicts of interest.
