# Supplementary material for: Causal associations between plasma metabolites and head and neck cancer: a bidirectional Mendelian randomization study
Source: Braz J Otorhinolaryngol. 2026 Apr 28;92(4):101812. doi: 10.1016/j.bjorl.2026.101812 (PMC13140025; doi:10.1016/j.bjorl.2026.101812)

**BJORL-D-25-00203**

**Material Supplementary**

**Table S1** STROBE-MR checklist of recommended items to address in reports of Mendelian randomization studies.

**Table S2** Detailed information of genetic datasets used in this study.

**Table S3** The list of GWAS data for 1400 plasma metabolites.

**Table S4** Detailed information of instrumental variables used in preliminary analysis.

**Table S5** Detailed information of instrumental variables used in replication analysis.

**Table S6** Detailed information of instrumental variables used in reverse preliminary analysis.

**Table S7** Detailed information of instrumental variables used in reverse replication analysis.

**Table S8** MR results of causal effects in preliminary analysis.

**Table S9** MR results of causal effects in replication analysis.

**Table S10** MR results of causal effects in reverse preliminary analysis.

**Table S11** MR results of causal effects in reverse replication analysis.

**Figure S1** Identified plasma metabolites passed the replication test.


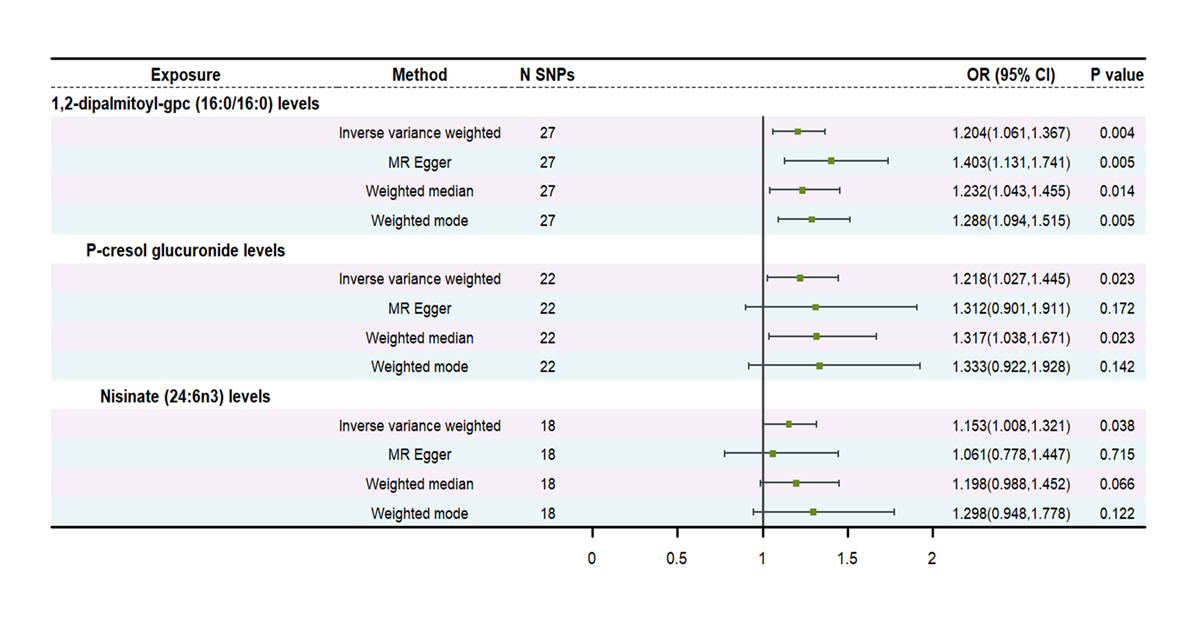


**Figure S2** The forest plots of preliminary analysis (A‒C) and replication analysis (D‒F).


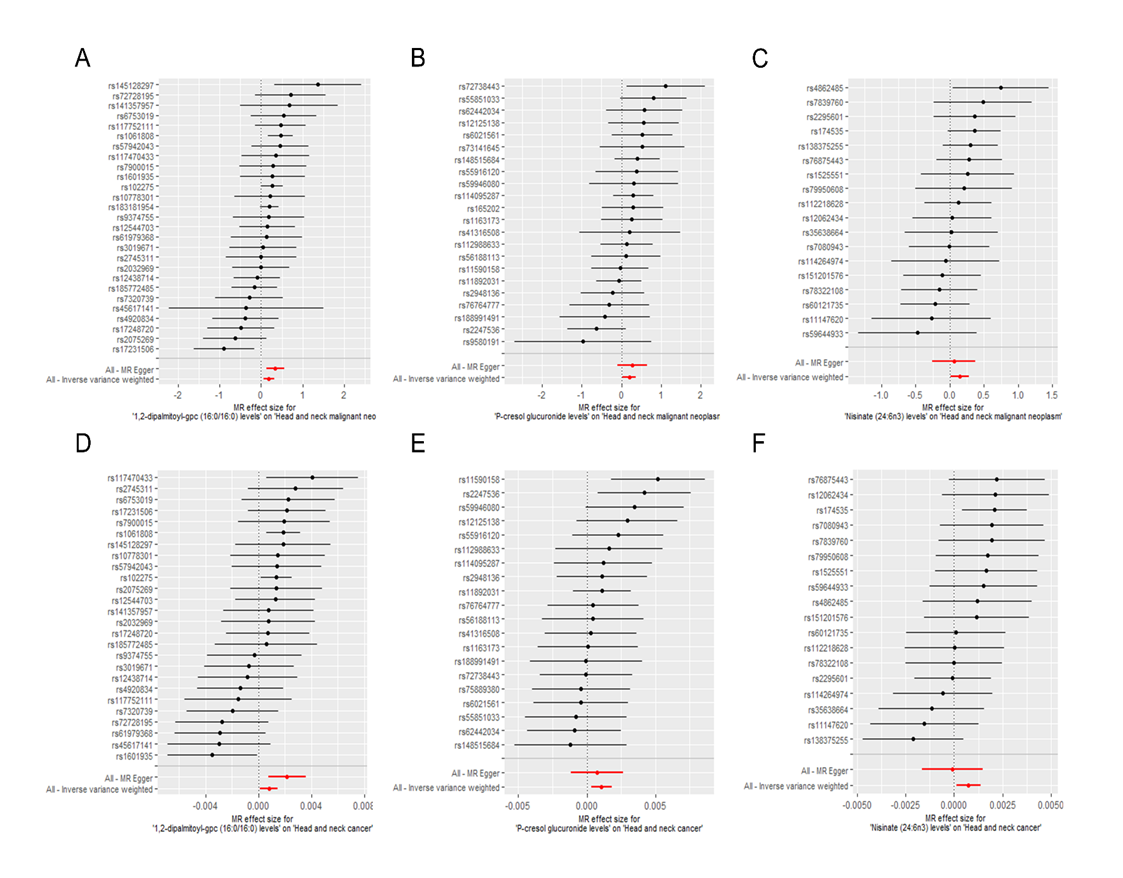


**Figure S3** The funnel plots of preliminary analysis (A‒C) and replication analysis (D‒F).


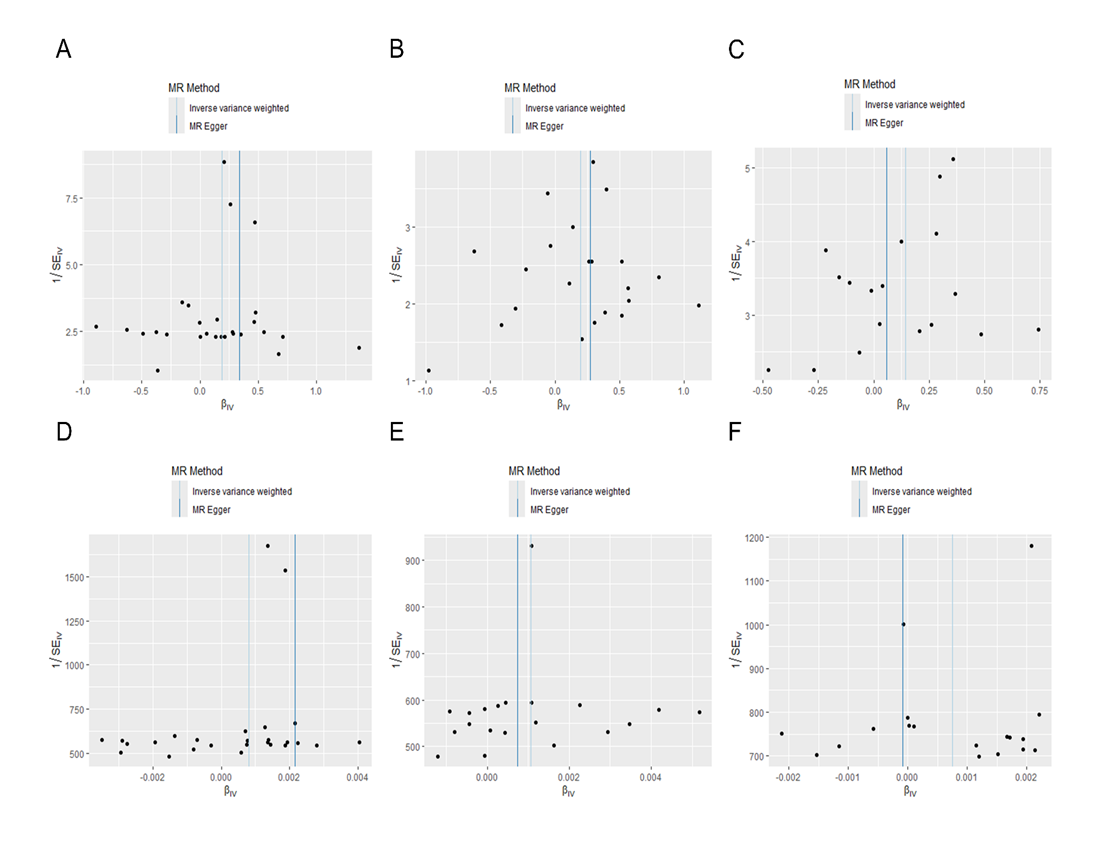

Supplement: Supplementary file 1 [file mmc1.docx]
